# Efficacy of melanoma patients treated with PD-1 inhibitors: Protocol for an overview, and a network meta-analysis of randomized controlled trials

**DOI:** 10.1097/MD.0000000000016342

**Published:** 2019-07-05

**Authors:** Jing Li, Shu-Zhen Shi, Jian-Shu Wang, Zhao Liu, Jin-Xu Xue, Jian-Cheng Wang, Jun-Hai Jia

**Affiliations:** a Gansu Provincial Cancer Hospital; b School of Basic Medical Sciences, Lanzhou University; c Gansu Provincial Hospital; d Hospital Management Research Center, Lanzhou University; e Gansu Province Hospital Rehabilitation Center, China.

**Keywords:** melanoma, network meta-analysis, overview, PD-1 inhibitors

## Abstract

**Background:**

Melanoma is a malignant tumor of melanocytes that produces pigments and can occur in the whole body. It is characterized by strong invasiveness, high metastasis rate and poor prognosis, and brings heavy burden to patients and society. In order to find the most effective and safe treatment measures, in this study, a network meta-analysis (NMA) for randomized controlled trials (RCTs) of advanced melanoma treated with PD-1 inhibitors will be conducted based on the existing systematic reviews (SRs) of PD-1 inhibitor in the treatment of advanced melanoma.

**Methods:**

PubMed, EMBASE, Web of Science and the Cochrane Library were searched on December 18, 2018 to obtain systematic reviews of PD-1 inhibitor in the treatment of advanced melanoma. Assessing the Methodological Quality of Systematic Reviews (AMSTAR2) will be used to assess the methodological quality of systematic reviews, Grading of Recommendations Assessment, Development, and Evaluation (GRADE) approach will be applied to evaluate the evidence quality of outcome measures, and the Cochrane's risk of bias tool will be utilized to appraise risks of bias of each embedded RCTs. And the outcomes are overall survival (OS), progression-free survival (PFS) and objective response rate (ORR). Hazard ratio (HR) or odds ratio (OR) with their 95% confidence interval (CI) were used to synthesize dichotomous outcomes, while the mean difference (MD) for the continuous variables. R3.5.1 will be used to create a network evidence map for direct and indirect comparative analysis.

**Results:**

This study will provide a comprehensive summary of the current evidences related to the efficacy and safety of PD-1 inhibitor in advanced melanoma.

**Conclusion:**

Our findings will be useful to assist clinicians make reasonable decisions to the treatment of advanced melanoma.

**Ethics and Communication:**

It is unnecessary for this NMA to acquire an ethical approval, because it is based on published researches.

**PROSPERO registration number:**

CRD42019120017

## Abstract 2

Supplemental Digital Content is available in the text

## 1 Introduction

Melanoma, also known as malignant melanoma, is a type of malignant tumor of melanocytes, originating from the neuroectoderm, which can produce pigment, and can occur throughout the body (including the skin, iris, and digestive tract), accounting for 75% of the deaths caused by malignant skin tumors, has characteristics of strong invasion, high metastasis and poor prognosis.^[1,2]^ Clinically, melanoma usually occurs between 40 and 60 years old, but it can still be seen in adolescence and old age. In women, they most commonly occur on the legs, while in men they are most common on the back. The average age of patients at diagnosis is 57 years old. Melanoma has the highest rate of brain metastasis among solid tumors in adults, and has traditionally been difficult to treat with all therapies, so brain metastasis often leads to or contributes to death.^[3]^ For advanced/unresectable melanoma, the prognosis until recently was very poor and treating physicians had limited effective therapeutic options.^[4]^ Skin melanoma is currently a major public health problem due to the rising incidence of melanoma worldwide. This growth rate is higher than any other cancers and is regarded as an epidemic.^[5]^ Treatment of advanced malignant melanoma is performed from a multidisciplinary approach, surgery, add on treatment, chemotherapy, targeted therapy, immunotherapy, radiation.^[6]^ In recent years, with the improvement of scientific research ability, the important role of the immune system in tumor control has been explored. Therefore, immunotherapy such as anti-PD-1 and anti-CTLA-4 has emerged. Some studies^[7,8]^ have found that Anti-PD-1 seems to have an increased response rate and more tolerable safety profile than anti-CTLA-4 in malignant melanoma/unresectable metastatic melanoma. In the absence of direct comparisons of all interventions, indirect treatment comparisons using network meta-analysis (NMA) from various randomized controlled trials (RCTs) can provide useful evidence for health care decision-making.^[9]^ In this paper, we will conduct a reanalysis for the systematic reviews (SRs) of advanced melanoma treated with PD-1 inhibitors, and an NMA of RCTs of PD-1 inhibitors in the treatment of advanced melanoma included in these SRs, in order to find the most effective and safe treatment measures.

## 2 Objectives

Based on the existing SRs of PD-1 inhibitor in the treatment of advanced melanoma, this study comprehensively analyzed the results of the effectiveness of existing SRs, summarize systematically the best current evidence on survival associated with PD-1 inhibitor in the treatment of advanced melanoma, and also an NMA of RCTs that included in the existing SRs will plan to be conducted, hoping to find the best treatment scheme for advanced melanoma.

## 3 Study methods and analysis

This protocol will be performed in accordance with recommendations of “Preferred Reporting Items for Systematic Review and Meta-Analysis Protocols” (PRISMA-P) statement guidelines. A study^[10]^ has shown that prospective registration can effectively improve the overall methodological quality of SRs, so current study has been registered on the International prospective register of systematic reviews (PROSPERO), the registration number is CRD42019120017, Available from http://www.crd.york.ac.uk/PROSPERO/display_record.php?ID=CRD42019120017

This is an overview and a NMA based on published studies; therefore, ethical approval is not required.

### 3.1 Eligibility criteria

#### 3.1.1 Participants

Patients with melanoma, include stage III or IV melanoma, unresectable metastatic melanoma, malignant melanoma, and advanced melanoma.

#### 3.1.2 Intervention

All types of PD-1 inhibitors, such as nivolumab, pembrolizumab, which used alone or in combination with other interventions.

#### 3.1.3 Comparator

Different PD-1 inhibitors, or same PD-1 inhibitor with different doses, or chemotherapy, or placebo, or without treatments, or others.

#### 3.1.4 Outcomes

We set overall survival (OS) as the primary outcome, and progression-free survival (PFS) and objective response rate (ORR) as secondary outcomes. OS (time between the start of treatment and death due to any cause); PFS (time between the start of treatment and documented disease progression or death due to any cause); ORR (the proportion of patients whose tumor has shrunk to a certain amount and remained for a certain period of time, including complete response (CR) or partial response (PR) cases)

#### 3.1.5 Design

To be included, SRs must include RCTs, meta-analysis results, and satisfy the participants, interventions, controls and outcomes of interest criteria described.

#### 3.1.6 Exclusion criteria

Duplicate records, conference papers, letters, the data is incomplete or unclear which is still unable to obtain after contacting the author, the older version of the updated SRs not used for supplemental data, only reported narratively data without meta-analysis results.

### 3.2 Data source

Literature search and review of relevant articles were limited to human studies. We searched the following electronic bibliographic databases: PubMed, EMBASE, Web of Science and the Cochrane Library for relevant articles published in English, without time restriction. All searches are until December 18, 2018. The search strategy combines medical subject headings (MeSH) and free words with “AND”,“OR” the two logical operators. In order to avoid missed inspections to obtain comprehensive search results, our search content mainly includes interventions and research design. The logical operator “OR” was used to connect the different interventions, the same to study design, but the “AND” was used to connect the interventions and the study design, the detailed search strategy in PubMed is available in Supplemental Digital Content (Appendix 1).

After the retrieval results were imported into Endnote8, the titles and abstracts of the retrieved literature were read by 2 researchers independently after computer deduplication. After the literature that obviously did not meet the inclusion criteria were excluded, the literature that might meet the inclusion criteria were read in full to determine. The included literature was cross-checked by 2 researchers. For the literature with differences and difficult to reach consensus, the inclusion was determined by discussion or by the third part. Then, RCTs included in eligible SRs will be obtained for NMA.

The data extraction form will be designed by 2 experienced reviewers. Three of the standards-compliant articles will be tested on the developed form. After full preparation, the same 2 reviewers will conduct data extraction independently. Any disagreement will be resolved by negotiation between the 2 parties, and if the agreement is still not reached, the third party will be required to decide. First, for each identified SRs, basic information (title, the first author, year of publication, journal, funding), PICOS, numbers of RCTs included in each SR, main outcomes, publication bias, and conclusions will be extracted. Second, the following data will be extracted from full text of each embedded RCTs: study identification, country, journal, funding, PICO, and other information.

### 3.3 Methodological quality assessment of included SRs

Recently more and more researchers use SRs to synthesize research evidence to address health issues at the global and national levels. However, due to the complexity and diversity of research in this field, the methodology of SRs has also been facing challenges.^[11]^ So, Assessing the Methodological Quality of Systematic Reviews (AMSTAR2),^[12]^ which is commonly used to assess the methodological quality of SRs will be utilized by two independent reviewers to reflect risk of bias or validity of included SRs process and results.^[13]^ The AMSTAR2 checklist has a total of 16 evaluation items. If the item is answered correctly and the basis is sufficient, the judgment is “Yes”; if the item is answered correctly but the basis is not sufficient, the judgment is “partially”; if the item has no relevant evaluation content or improper evaluation, the judgment is “No”. For each item, the answer is “yes” for 1 point, “partial” for 0.5 points, and the rest of the evaluation results for 0 points for a total of 16 points. The final AMSTAR2 checklist score of 0 to 3 is considered to be low quality, 4 to 7 points are considered to be low quality, 8 to 11 points are considered to be medium quality, and 12 to 16 points are considered to be of high quality.

If there is any difference, the 2 reviewers will discuss together and solve it. If there is still no consensus reached, the third party should be invited to make a decision.

### 3.4 Evidence quality of outcome measures

Grading of Recommendations Assessment, Development, and Evaluation (GRADE)^[14]^ approach will be used to evaluate the evidence quality of outcome measures. The level of evidence contains 4 grades as follows: very low (We have very little confidence in the effect estimate: The true effect is likely to be substantially different from the estimate of effect), low (Our confidence in the effect estimate is limited: The true effect may be substantially different from the estimate of the effect), moderate (We are moderately confident in the effect estimate: The true effect is likely to be close to the estimate of the effect, but there is a possibility that it is substantially different), and high (We are very confident that the true effect lies close to that of the estimate of the effect). At the very beginning, the quality of evidence of all outcomes was classified as “high” by default, and after rating, each outcome could receive a quality grade of high, moderate, low, or very low.^[15]^

### 3.5 Assessment of risk of bias of included RCTs in identified SRs

For each embedded RCTs, their risks of bias will be assessed by the Cochrane's risk of bias tool.^[16]^ The evaluation tool includes 6 aspects:

1. selection bias: random sequence generation, allocation concealment;
2. performance bias: Blinding of participants and personnel;
3. detection bias: Blinding of outcome assessment;
4. Incomplete outcome data: result data integrity;
5. Reporting bias: selective reporting;
6. Other biases: other important biases that do not include the above. For each result, “Low” (Low bias risk), “High” (High bias risk) and “Unclear” (Uncertain bias or lack of relevant information) will be made based on the above six evaluation criteria.

### 3.6 Dealing with missing data

If the data is incomplete or missing, we will contact the author by email for information.

### 3.7 Data synthesis

#### 3.7.1 Basic characteristics

We will provide a descriptive analysis of the basic characteristics of the included SRs.

#### 3.7.2 Evidence map

The bubble plot will be produced according to the methodological quality, where each bubble represents one SR. The information of 3 dimensions in the map are

1. the bubble size represents the number of primary studies included in the SRs,
2. the methodological quality in the x-axis,
3. the interventions in the y-axis.

#### 3.7.3 Network meta-analyses of included RCTs

We will use R3.5.1 to create a network evidence map for direct and indirect comparative analysis. Hazard ratio (HR) or odds ratio (OR) with their 95% confidence interval (CI) were used to synthesize dichotomous outcomes, while the mean difference (MD) for the continuous variables. *P* < .05 was considered statistically significant. Heterogeneity analysis was performed on the included studies in the combined analysis, and the I^2^ value represented the size of the heterogeneity. If I^2^ < 25% or < 50% respectively indicates that the heterogeneity is low or moderate, the fixed effect model will be used to combine analysis; If I^2^ ≥50% indicates a high heterogeneity, we will further analyze whether it is clinical or methodological. After excluding clinical heterogeneity, a random effects model will be used to perform meta-analysis. Significant heterogeneity is treated using subgroup analysis or sensitivity analysis, or only descriptive analysis. Qualitative analysis will be conducted if the quantitative analysis is not possible. For a closed loop, a node-splitting model is used to detect inconsistency between direct and indirect comparisons.^[17]^ When *P* > .05, it is considered to be consistent, the consistency model could be used in NMA. Otherwise, the inconsistency model will be used. After comparing various interventions, the surface under the cumulative ranking (SUCRA) was calculated to rank the advantages and disadvantages of the interventions according to the SUCRA value (the greater the value, the better the intervention).^[18]^ The degree of convergence of the model was evaluated by the Brooks–Gelman–Rubin (BGR) method with the potential scale reduction factor (PSRF). PSRF values close to 1 indicate better convergence effect of the model, and generally, PSRF values less than 1.2 are acceptable.^[19]^

### 3.8 Sensitivity analysis

If necessary, sensitivity analysis should be carried out to determine the stability of the meta-analysis results by excluding high-quality or low-quality studies, major weight studies, and small studies. If the meta-analysis results are not significantly changed from the previous ones, it indicates that the results are stable and reliable.

### 3.9 Subgroup analysis

Subgroup analyses will be performed based on different PD-1 inhibitors, or PD-L1 positive/negative, or BRAF wild-type/BRAF mutant-positive patients if necessary data available.

### 3.10 Publication bias

Because the funnel plot judges the publication bias based on the number of studies that are greater or less than the combined effect, when the inclusion of the study is rare, the results are easily affected by the number of not included studies, so when we perform NMA, Only when the number of RCTs included is greater than 10, we can perform publication bias analysis by symmetry of the inverted funnel plot.

## 4 Results

**Figure 1:**
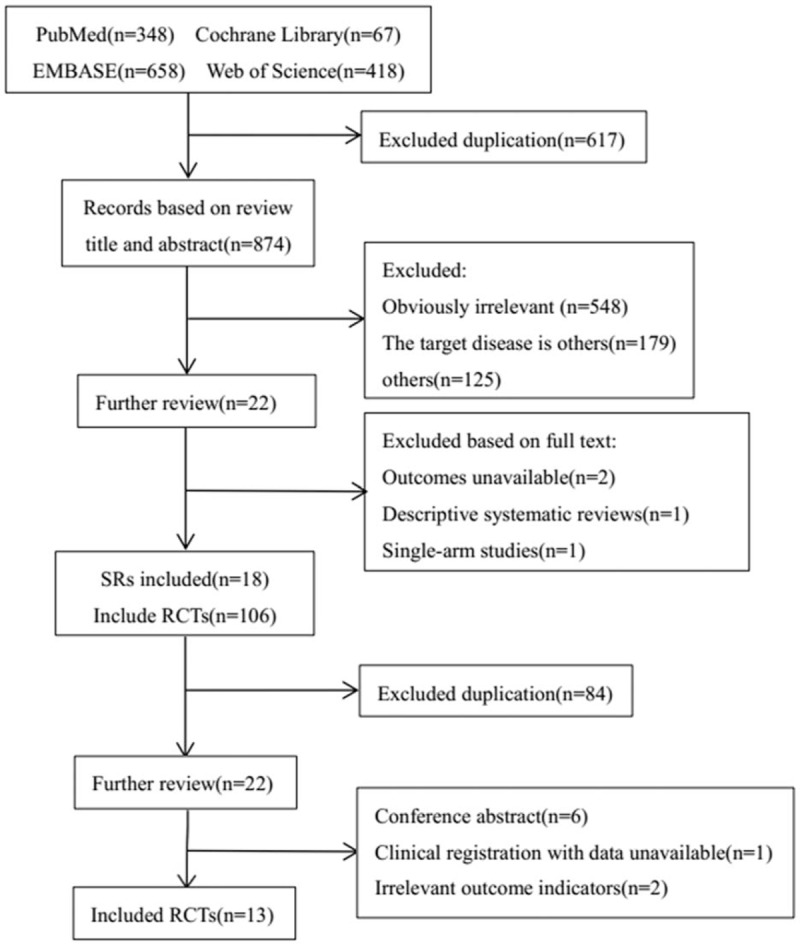
A flow diagram of the literature search and selection process.

In the present study, we included 18 SRs and collected 13 RCTs. We have searched PubMed (n = 348), Cochrane Library (n = 67), EMBASE (n = 658), Web of Science (n = 418) these 4 English databases and retrieved a total of 1491 records. After removing duplicates, a total of 874 records remained. After screening based on title and abstract, 852 records were excluded and only 22 records for further review. By finding and reading the full text, finally 18 SRs were identified, and they contained 106 RCTs. Similar to the SR screening step, excluded duplications (n = 84) and full text unavailable or inappropriate (n = 9), finally 13 RCTs were included. The complete process is presented in a PRISMA flow diagram (Fig. 1).

## 5 Discussions

Melanoma is one of the most aggressive cancers caused by malignant transformation of melanocytes, the United States had an estimated 73,870 new cases and nearly 10,000 melanoma deaths, accounting for nearly 75 percent of all skin cancer deaths in 2015.^[20,21]^ Melanocytic neoplasms are both common lesions in clinical practice and a frequent source of diagnostic difficulty for the general surgical pathologist.^[22]^ For most newly diagnosed melanoma patients, surgical resection is effective in most cases.^[23]^ But about 10% of melanoma cases are diagnosed at an advanced stage and cannot be removed or metastasized. In stage IV tumors, approximately one-third of patients have visceral and cerebral involvement at diagnosis, with a poor prognosis and a low likelihood of sustained response to treatment.^[24]^ The FDA approved 10 new treatments for metastatic melanoma between 2011 and 2015, which is unprecedented and exciting. Melanoma patients and their doctors now have many treatment options available.^[25]^ Although currently there are more options for the treatment of melanoma. However, in order to further optimize the treatment regimen and improve the quality of life of patients, more research in related fields is still needed.

## Author contributions

**Data curation:** Jian-Shu Wang, Zhao Liu, Jin-Xu Xue.

**Formal analysis:** Jing Li, Shu-Zhen Shi.

**Project administration:** Jun-Hai Jia.

**Writing – original draft:** Jing Li, Shu-Zhen Shi, Jian-Cheng Wang.

## Supplementary Material

Supplemental Digital Content
