# Supplementary material for: Efficacy of melanoma patients treated with PD-1 inhibitors: Protocol for an overview, and a network meta-analysis of randomized controlled trials
Source: Medicine (Baltimore). 2019 Jul 5;98(27):e16342. doi: 10.1097/MD.0000000000016342 (PMC6635302; doi:10.1097/MD.0000000000016342)
Supplement: Supplemental Digital Content [file medi-98-e16342-s001.docx]

PubMed

#1 nivolumab [Supplementary Concept] OR MDX-1106[Title/Abstract] OR ONO-4538[Title/Abstract] OR BMS-936558[Title/Abstract] OR Opdivo[Title/Abstract] OR nivolumab[Title/Abstract] OR bms 936558[Title/Abstract] OR bms936558[Title/Abstract] OR mdx 1106[Title/Abstract] OR mdx1106[Title/Abstract] OR ono 4538[Title/Abstract] OR ono4538[Title/Abstract]

#2 pembrolizumab [Supplementary Concept] OR Pembrolizumab[Title/Abstract] OR MK-3475[Title/Abstract] OR Keytruda[Title/Abstract] OR Lambrolizumab[Title/Abstract] OR IBI308[Title/Abstract] OR mk 3475[Title/Abstract] OR mk3475[Title/Abstract]

#3 avelumab [Supplementary Concept] OR Avelumab[Title/Abstract] OR bavencio[Title/Abstract] OR msb 0010682[Title/Abstract] OR msb 0010718c[Title/Abstract] OR msb 10682[Title/Abstract] OR msb 10718c[Title/Abstract] OR msb0010682[Title/Abstract] OR msb0010718c[Title/Abstract] OR msb10682[Title/Abstract] OR msb10718c[Title/Abstract]

#4 atezolizumab [Supplementary Concept] OR Atezolizumab[Title/Abstract] OR monoclonal antibody mpdl 3280a[Title/Abstract] OR monoclonal antibody mpdl3280a[Title/Abstract] OR mpdl 3280a[Title/Abstract] OR mpdl3280a[Title/Abstract] OR rg 7446[Title/Abstract] OR rg7446[Title/Abstract] OR tecentriq[Title/Abstract] OR tecntriq[Title/Abstract] OR MPDL3280A[Title/Abstract] OR RG-7446[Title/Abstract]

#5 Nivolizumab[Title/Abstract]

#6 durvalumab [Supplementary Concept] OR Durvalumab[Title/Abstract] OR MEDI4736[Title/Abstract] OR MEDI-4736[Title/Abstract] OR Imfinzi[Title/Abstract] OR medi 4736[Title/Abstract]

#7 "pidilizumab" [Supplementary Concept] OR CT-011[Title/Abstract] OR CT 011[Title/Abstract] OR ct011[Title/Abstract]

#8 B7-H1 Antigen[Mesh] OR B7-H1 Antigen[Title/Abstract] OR B7 H1 Antigen[Title/Abstract] OR Programmed Cell Death 1 Ligand 1[Title/Abstract] OR B7-H1 Immune Costimulatory Protein[Title/Abstract] OR B7 H1 Immune Costimulatory Protein[Title/Abstract] OR PD-L1 Costimulatory Protein[Title/Abstract] OR PD L1 Costimulatory Protein[Title/Abstract] OR Programmed Cell Death 1 Ligand 1 Protein[Title/Abstract] OR CD274 Antigen[Title/Abstract] OR CD274 Antigens[Title/Abstract] OR B7H1 Immune Costimulatory Protein[Title/Abstract] OR antigen B7 H1[Title/Abstract] OR antigen B7H1[Title/Abstract] OR antigen CD274[Title/Abstract] OR B7 H1 protein[Title/Abstract] OR B7 homolog 1 protein[Title/Abstract] OR B7-H1 antigen[Title/Abstract] OR B7H1 antigen[Title/Abstract] OR B7H1 protein[Title/Abstract] OR PDCD1 ligand 1[Title/Abstract] OR PDCD1LG1 protein[Title/Abstract] OR protein B7 H1[Title/Abstract] OR protein B7H1[Title/Abstract] OR protein PDCD1LG1[Title/Abstract] OR programmed death ligand 1[Title/Abstract] OR programmed deathligand 1[Title/Abstract] OR programmed death 1 ligand 1 antibody[Title/Abstract] OR programmed death 1 ligand 1 protein[Title/Abstract] OR programmed cell death Ligand-1[Title/Abstract] OR programmed cell death-Ligand 1[Title/Abstract] OR programmed cell death-Ligand-1[Title/Abstract] OR programmed cell death 1 ligand 1[Title/Abstract] OR PDL1[Title/Abstract] OR PD-L1[Title/Abstract] OR PD-L1[Title/Abstract] OR PD L1[Title/Abstract] OR PD 1[Title/Abstract] OR PD1[Title/Abstract] OR PD-1 antibody[Title/Abstract] OR PD-1[Title/Abstract] OR programmed cell death 1[Title/Abstract] OR programmed cell death-1[Title/Abstract] OR programmed death 1 receptor[Title/Abstract] OR programmed death 1 receptor antibody[Title/Abstract] OR programmed cell death 1 receptor[Title/Abstract]

#9 OR/1-8

#10 Network Meta-Analysis[Mesh] OR network meta analysis[Title/Abstract] OR network meta analyses[Title/Abstract]

#11 network meta-analysis[Title/Abstract] OR network meta-analyses[Title/Abstract] OR network metaanalyses[Title/Abstract] OR network metaanalysis[Title/Abstract] OR mixed treatment comparison meta analysis[Title/Abstract] OR mixed treatment comparisons meta analyses[Title/Abstract] OR mixed treatment meta analysis[Title/Abstract] OR mixed treatment meta analyses[Title/Abstract] OR mixed treatment comparison meta-analysis[Title/Abstract] OR mixed treatment comparisons meta-analyses[Title/Abstract] OR mixed treatment meta-analysis[Title/Abstract] OR mixed treatment meta-analyses[Title/Abstract] OR mixed treatment comparison metaanalysis[Title/Abstract] OR mixed treatment comparisons metaanalyses[Title/Abstract] OR mixed treatment metaanalysis[Title/Abstract] OR mixed treatment metaanalyses[Title/Abstract] OR multiple treatment comparison meta analysis[Title/Abstract] OR multiple treatment comparisons meta analyses[Title/Abstract] OR multiple treatments meta analysis[Title/Abstract] OR multiple treatments meta analyses[Title/Abstract] OR multiple treatment meta analysis[Title/Abstract] OR multiple treatment meta analyses[Title/Abstract] OR multiple treatment comparison meta-analysis[Title/Abstract] OR multiple treatment comparisons meta-analyses[Title/Abstract] OR multiple treatments meta-analysis[Title/Abstract] OR multiple treatments meta-analyses[Title/Abstract] OR multiple treatment meta-analysis[Title/Abstract] OR multiple treatment meta-analyses[Title/Abstract] OR multiple treatment comparison metaanalysis[Title/Abstract] OR multiple treatment comparisons metaanalyses[Title/Abstract] OR multiple treatments metaanalysis[Title/Abstract] OR multiple treatments metaanalyses[Title/Abstract] OR multiple treatment metaanalysis[Title/Abstract] OR indirect comparison[Title/Abstract] OR indirect treatment[Title/Abstract] OR indirect comparisons[Title/Abstract] OR indirect treatments[Title/Abstract]

#12 meta analysis[Title/Abstract] OR meta analyses[Title/Abstract] OR metaanalysis[Title/Abstract] OR metanalysis[Title/Abstract] OR met-analysis[Title/Abstract] OR metaanalyses[Title/Abstract] OR metanalyses[Title/Abstract] OR met-analyses[Title/Abstract] OR systematic review[Title/Abstract] OR systematic reviews[Title/Abstract]

#13 "Meta-Analysis as Topic"[Mesh] OR "Meta-Analysis"[Publication Type]

#14 OR/10-13

#15 #9 AND #14
